# RN181 is a tumour suppressor in gastric cancer by regulation of the ERK/MAPK–cyclin D1/CDK4 pathway

**DOI:** 10.1002/path.5246

**Published:** 2019-04-11

**Authors:** Suihai Wang, Xiaobo Wang, Yanjun Gao, Yingxia Peng, Ningning Dong, Qian Xie, Xian Zhang, Yingsong Wu, Ming Li, Ji‐Liang Li

**Affiliations:** ^1^ Institute of Antibody Engineering, School of Laboratory Medicine and Biotechnology Southern Medical University Guangzhou PR China; ^2^ Wenzhou Medical University School of Biomedical Engineering and Eye Hospital Wenzhou Institute of Biomaterials and Engineering Wenzhou PR China; ^3^ Institute of Translational and Stratified Medicine University of Plymouth Faculty of Medicine and Dentistry Plymouth UK

**Keywords:** RN181, gastric carcinoma, tumour suppressor, cyclin D/CDK4, ERK1/2

## Abstract

RN181, a RING finger domain‐containing protein, is an E3 ubiquitin ligase. However, its biological function and clinical significance in cancer biology are obscure. Here, we report that RN181 expression is significantly down‐regulated in 165 tumour tissues of gastric carcinoma (GC) versus adjacent non‐tumour tissues, and inversely associated with tumour differentiation, tumour size, clinical stage, and patient's overall survival. Alterations of RN181 expression in GC cells by retrovirus‐transduced up‐regulation and down‐regulation demonstrated that RN181 functions as a tumour suppressor to inhibit growth of GC in both *in vitro* culture and *in vivo* animal models by decreasing tumour cell proliferation and increasing tumour cell apoptosis. Cell cycle analysis revealed that RN181 controls the cell cycle transition from G1 to S phase. Mechanistic studies demonstrated that RN181 inhibits ERK/MAPK signalling, thereby regulating the activity of cyclin D1–CDK4, and consequently controlling progression in the cell cycle from G1 to S phase. Restoring CDK4 in GC cells rescued the inhibitory phenotype produced by RN181 *in vitro* and *in vivo*, suggesting a dominant role of CDK4 in control of the tumour growth by RN181. Importantly, RN181 expression is inversely correlated with the expression of cyclin D1 and CDK4 in GC clinical samples, substantiating the role of the RN181–cyclin D1/CDK4 pathway in control of the tumour growth of GC. Our results provide new insights into the pathogenesis and development of GC and rationale for developing novel intervention strategies against GC by disruption of ERK/MAPK–cyclin D1/CDK4 signalling. In addition, RN181 may serve as a novel biomarker for predicting clinical outcome of GC. © 2019 The Authors. *The Journal of Pathology* published by John Wiley & Sons Ltd on behalf of Pathological Society of Great Britain and Ireland.

## Introduction

Gastric adenocarcinoma (gastric cancer, GC) is the fifth most common cancer and the third leading cause of cancer mortality worldwide 1. In China, however, GC is the second most common cancer and the second leading cause of cancer death, having estimated numbers of 679 100 new cases and 498 000 deaths in 2015 2. Most GC patients present with advanced or metastatic disease in clinics and are treated only by palliative chemotherapy, with an overall 5‐year survival around 5–20% and a median overall survival of 11–12 months 3. In addition to standard perioperative chemotherapy or postoperative chemoradiation 4, 5, 6, targeted therapy, which targets specific molecules and disrupts the activity of a specific oncogenic signalling pathway, has emerged as a promising therapeutic strategy. Recently, a randomised phase 3 trial for the first‐line treatment of HER2‐positive advanced GC showed that trastuzumab, a humanised monoclonal antibody targeting HER2, when combined with standard chemotherapy, significantly improves an overall survival of GC patients from 11.1 months to 13.8 months 3. Unfortunately, only 7–34% of GC patients are HER2‐positive 3, 7 and thus clinical application of trastuzumab is highly restricted. Therefore, it is imperative that identification of new targets will help to understand the molecular mechanisms of pathogenesis and develop a novel targeted therapy or biomarker for evaluating the therapeutic efficacy or predicting the prognosis of GC patients 8.

RN181 (also named RNF181 or HSPC238) is a member of the really interesting new gene (RING) finger protein family whose members are well recognised in protein dimerisation and protein–protein interaction and possess ubiquitin ligase activities 9. It was reported that in platelets, RN181 interacts with the cytoplasmic regulatory domain of integrin αIIbβ3 and exhibits E3 ubiquitin ligase activity for auto‐ubiquitination 10. However, the physiological substrates and biological functions of RN181 in cancer biology are obscure. We have been interested in RING finger ubiquitin ligases in cancer biology and clinical significance. In this paper, we report that RN181 is significantly down‐regulated in GC clinical samples and is closely associated with some clinicopathological features and patient's overall survival. Up‐regulation and down‐regulation of RN181 demonstrated that RN181 functions as a tumour suppressor that controls the tumour growth of GC. Mechanistic studies revealed that RN181 controls tumour growth through induction of cell cycle arrest at the G1–S phase transition by inhibition of ERK/MAPK signalling and thereby regulation of the cyclin D1–CDK4 activity in GC.

## Materials and methods

### Cell culture

The lentivirus packaging cell line (GP2‐293) and GC cell lines (AGS, MKN28, and MKN45) were purchased from and authenticated by the Shanghai Institutes for Biological Sciences (Shanghai, PR China). All cell lines were cultured in Dulbecco's modified Eagle's medium (Gibco, Grand Island, NY, USA) supplemented with 10% fetal bovine serum, 100 U/ml penicillin, and 100 μg/ml streptomycin, and maintained at 37°C with 5% CO_2_.

### Patients and tissue samples

GC clinical samples (HStm‐Ade167Sur and HStm‐Ade180Sur) containing clinicopathological information were purchased from Shanghai Outdo Biotech Co, Ltd (http://www.superchip.com.cn/). One hundred and sixty‐five GC tissue specimens paired with adjacent non‐tumour gastric tissues were used for immunohistochemical (IHC) studies. Patients' consent and approval from local Ethics Committee were obtained for research in use of clinical materials. The patients included 118 males and 47 females, with a median age of 65 years (range 34–84 years) (supplementary material, Table S1). Clinical stages were according to the 7th edition of the *AJCC Cancer Staging Manual* 11. Another cohort (HStm‐Ade150CS‐01) of clinical samples, consisting of 75 pairs of GC and adjacent non‐tumour tissues, was used for analysis of the expression correlation between RN181, cyclin D1, and CDK4.

### Tumour xenografts

BALB/c female nude mice 4–5 weeks of age were purchased from the Experimental Animal Center, Southern Medical University (Guangzhou, PR China) and maintained under standard pathogen‐free conditions. Tumour xenografts were performed as described previously 12. The left flank of each mouse was implanted with control tumour cells, while the right side was injected with the test tumour cells. Each group contained six mice. Tumour growth was monitored by measuring tumour length and width with callipers. Tumour volume was calculated with the formula [length × width^2^ × (π/6)] 13. All experimental procedures were approved by the Ethical Committee of Southern Medical University.

### Immunohistochemical staining and scoring

IHC staining was performed as described previously 12. Histopathological features of stained tumour were assessed by two researchers who were blinded to the patient's clinical characteristics. The intensity of RN181 immunostaining of GC tissues was scored as negative (0), weak (1), medium (2) or strong (3). The extent of staining, defined as the per cent of positive staining cells, was scored as 1 (≤ 10%), 2 (11–50%), 3 (51–75%) or 4 (> 75%). An overall expression score for statistical analysis, ranging from 0 to 12, was calculated by multiplying the score of intensity and that of extent. The final staining score was presented as low (overall score of ≤ 3) or high (overall score of > 3). Nuclear localisation of cyclin D1 or CDK4 was quantified by counting approximately 500 stained cells under 400× magnification.

IHC staining of anti‐RN181 (1:300 dilution), anti‐Ki67 (1:200 dilution), anti‐activated caspase 3 (1:100 dilution), anti‐p21 (1:400 dilution), anti‐cyclin D1 (1:400 dilution), anti‐CDK4 (1:400 dilution) and anti‐pERK1/2 (Thr202/Tyr204) (1:400 dilution) (#4370S; Cell Signaling, Danvers, MA, USA) for xenograft tumour sections was performed and scored as described above.

### Statistics

SPSS 13.0 software (SPSS Inc, Chicago, IL, USA) was used. The analysis of variance (ANOVA) test was used to compare mean values among three or more groups, whereas independent‐sample Student's *t*‐test was used to compare two groups with normal distribution data. The data normality was verified using the Kolmogorov–Smirnov test. For non‐normal distribution data, the Mann–Whitney test was used for two‐group comparisons, while the Jonckheere–Terpstra test was used to compare more than two groups. Sample‐paired *t*‐tests were employed to analyse the expression of RN181 in paired clinical samples and xenograft tumour sizes. Kaplan–Meier plots and the log‐rank test were used for analysis of overall survival data. Univariate and multivariate survival analyses were conducted using the Cox proportional hazards regression model. The Pearson correlation test was used to analyse the correlation between the expression of two proteins. Statistical significance is indicated in the figures by an asterisk where *p* < 0.05 and by two asterisks where *p* < 0.01.

## Results

### RN181 is down‐regulated and associated with overall survival of GC patients

**Figure 1:**
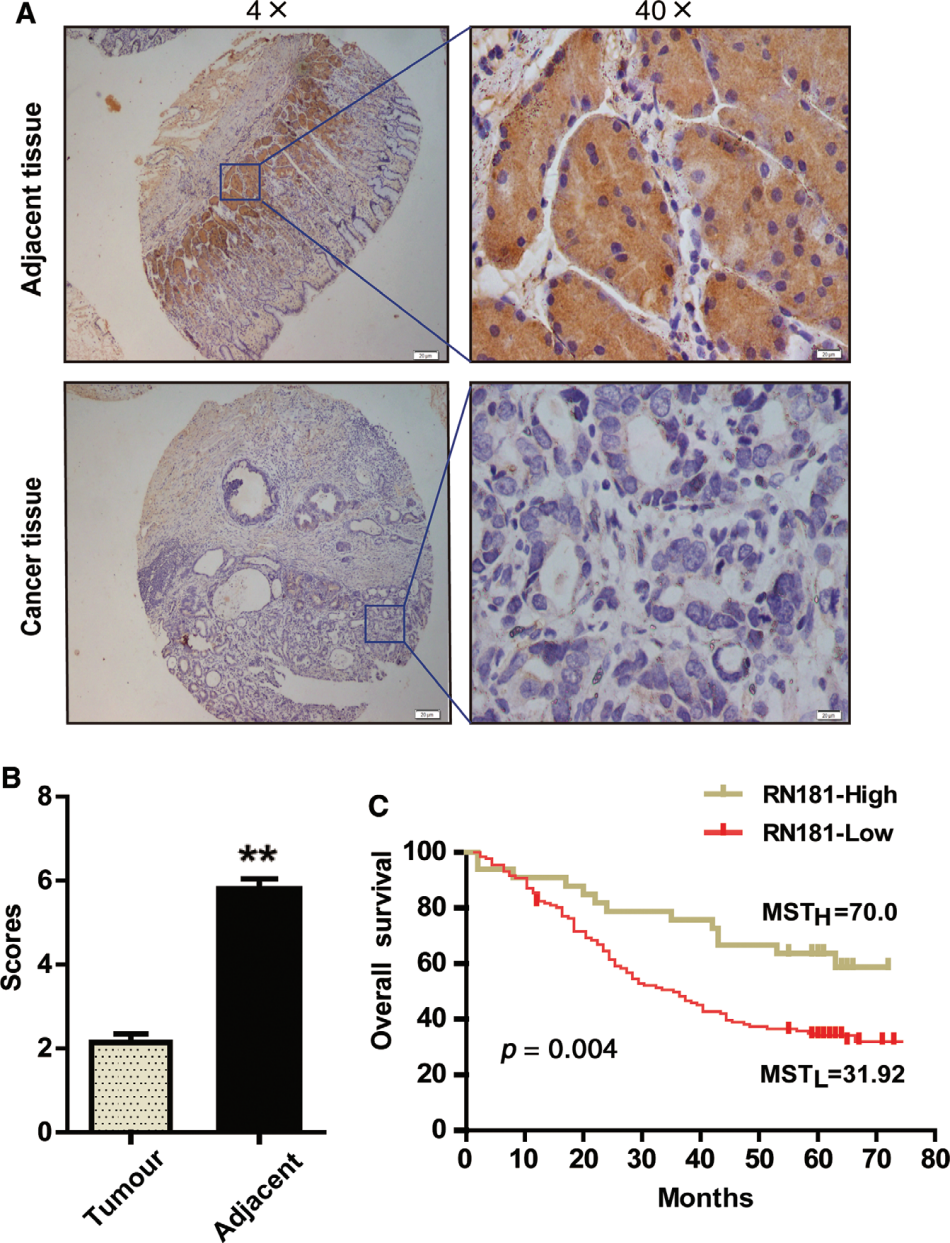
RN181 is down‐regulated and associated with overall survival in GC patients. (A) Representative images of RN181 expressed in GC tumour samples and adjacent non‐tumour tissues examined by IHC. (B) Quantification of IHC scores of RN181 in GC tumour samples and adjacent non‐tumour tissues. The data (mean ± SE, *n* = 165) were analysed by sample‐paired *t*‐test. **p < 0.01. (C) Kaplan–Meier survival analysis comparing GC patients with tumours having high and low expression of RN181. Median survival time of the high expression group (MST_H_ > 70.00) versus that of the low expression group (MST_L_ = 31.92). *n* = 165; **p < 0.01.

We examined the expression of RN181 in a cohort of 165 pairs of GC and adjacent non‐tumour tissues by IHC (Figure 1A and supplementary material, Figure S1). Quantitative analysis by scoring the staining showed that RN181 was significantly down‐regulated in tumour tissues versus adjacent tissues (2.19 ± 2.10 versus 5.94 ± 4.22) (*p* < 0.01) (Figure 1B). Clinicopathological analyses demonstrated that the expression of RN181 was significantly associated with tumour differentiation (*Z* = −2.045, *p* = 0.041), tumour size (*Z* = −2.484, *p* = 0.013), and clinical stage (*Z* = −2.765, *p* = 0.006) but not with patient's gender or age (supplementary material, Table S1). Decreased expression of RN181 was frequently observed in tumours that were poorly differentiated, had a larger tumour size, and were in a late clinical stage. Kaplan–Meier analysis revealed that expression of RN181 was significantly associated with overall survival of GC patients (*p* = 0.004), showing a median survival of more than 70.0 months in the RN181‐high expression group versus 31.9 months in the RN181‐low expression group (Figure 1C). Univariate Cox regression analysis showed that many parameters including age, tumour size, metastasis, and clinical stage were significantly correlated with patient overall survival (Table 1). Multivariate analysis demonstrated that RN181 expression has a prognostic role (HR = 2.337, 95% CI 1.238–4.422, *p* = 0.009). Thus, the results suggest that down‐regulation of RN181 may be involved in the tumour development of GC and serve as an independent poor prognostic biomarker for GC patients.

**Table 1:** Univariate and multivariate Cox regression analysis of potential prognostic factors in 165 gastric cancer patients (HR: hazard ratio; CI: confidence interval)

Factors	Univariate analysis			Multivariate analysis		
	HR	95% CI	*P* value	HR	95% CI	*P* value
Gender (male/female)	1.410	0.705–1.659	0.720	1.410	0.887–2.242	0.146
Age (≤ 50/51–70/≥ 71 years)	1.572	1.126–2.196	0.008	1.843	1.303–2.607	0.001
Tumour size (≤ 2.5/2.5–6.5/≥ 6.5 cm)	1.581	1.152–2.170	0.005	1.400	0.990–1.979	0.057
Metastasis (no/yes)	0.373	0.192–0.723	0.004	1.981	0.986–3.981	0.055
Clinical stage (I/II/III/IV)	2.043	1.425–2.929	0.000	1.907	1.285–2.830	0.001
Differentiation (well/moderate/poor)	1.456	0.997–2.125	0.052	1.275	0.845–1.924	0.247
RN181 expression (low/high)	2.246	1.252–4.029	0.007	2.337	1.238–4.411	0.009

### RN181 suppresses tumour growth of GC in vitro and in vivo

**Figure 2:**
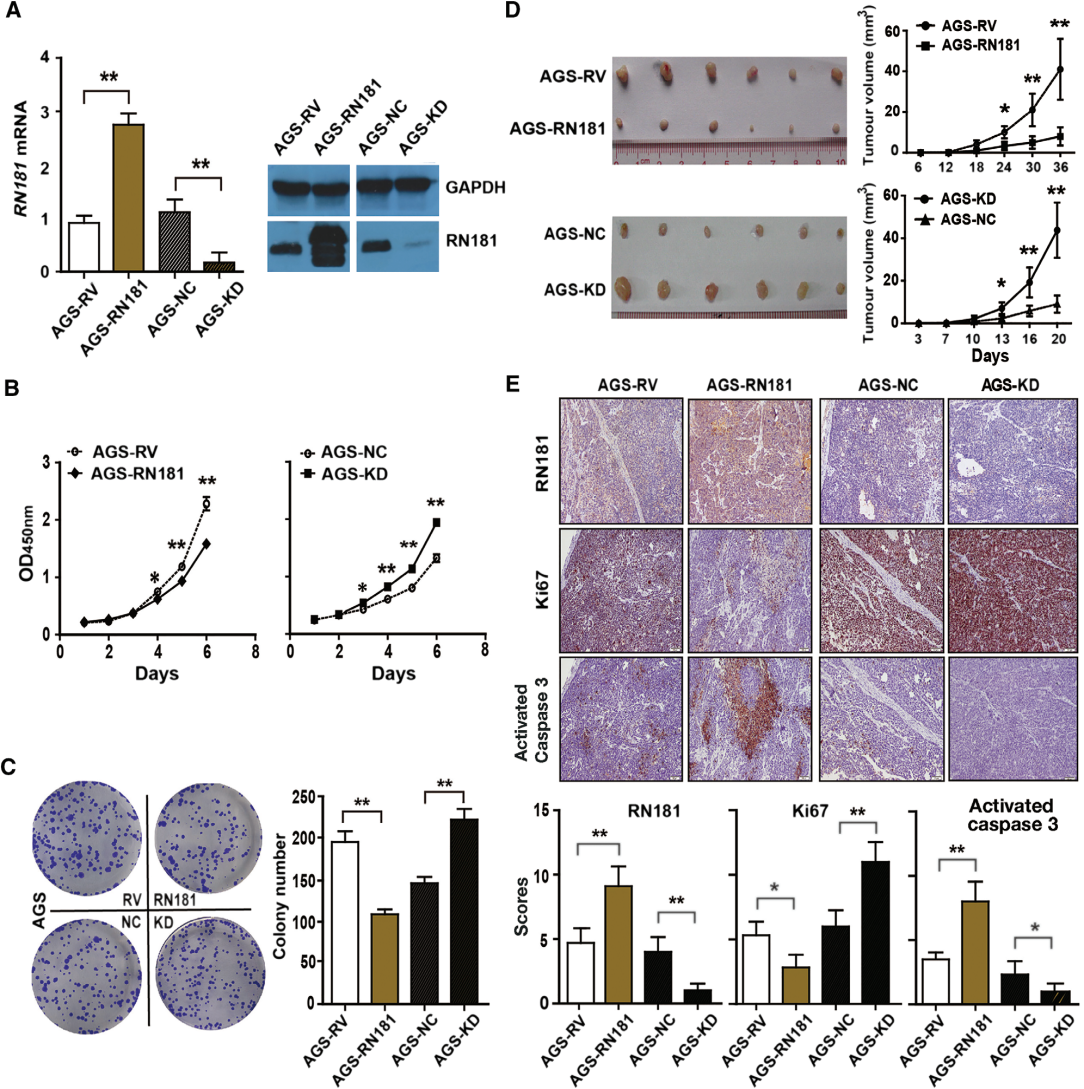
RN181 suppresses tumour growth of GC *in vitro* and *in vivo*. (A) Lentivirus‐mediated transduction to modulate the expression of RN181 in AGS cells at mRNA and protein levels as determined by QRT‐PCR (left panel) and western blotting (right panel), respectively. (B) Cell proliferation assays showed that up‐regulation of RN181 inhibited the growth (left panel), while down‐regulation of RN181 increased the growth of AGS cells (right panel) *in vitro*. (C) Colony formation assays showed that up‐regulation of RN181 decreased the number of colonies, whereas down‐regulation of RN181 increased the number of colonies formed by AGS cells *in vitro*. Mean ± SD, *n* = 3. *p < 0.05; **p < 0.01. (D) Up‐regulation of RN181 inhibited tumour growth (top panels), while down‐regulation of RN181 promoted tumour growth (bottom panels) of AGS cells in BALB/c nude mice. The data (mean ± SD, *n* = 6) were analysed by sample‐paired *t*‐test. *p < 0.05; **p < 0.01. (E) IHC and quantification of scoring for RN181, Ki67, and activated caspase 3 on xenograft sections of AGS cells. Original magnification ×10. The data (mean ± SD, *n* = 6) were analysed using Student's *t*‐test. *p < 0.05; **p < 0.01.

We up‐regulated the expression of RN181 in GC cells by retrovirus‐mediated transduction (Figure 2A). Cell proliferation assays demonstrated that up‐regulation of RN181 significantly inhibited the AGS growth (AGS‐RN181 versus AGS‐RV control, *p* < 0.01) (Figure 2B). Colony formation assays showed that up‐regulation of RN181 significantly reduced the number of colonies formed by AGS cells (AGS‐RN181 versus AGS‐RV control, *p* < 0.01) (Figure 2C). Consistently, down‐regulation of endogenous RN181 by transduction of shRNA‐containing viruses significantly increased the proliferation and colony formation of AGS cells (AGS‐KD versus AGS‐NC control, *p* < 0.01). Similar results were obtained from MKN28 and MKN45 cell lines in which RN181 expression was down‐regulated (supplementary material, Figure S2A–C).

We then implanted the GC cells into nude mice. Up‐regulation of RN181 significantly inhibited the tumour growth (AGS‐RN181 versus AGS‐RV, *p* < 0.01), while down‐regulation of RN181 dramatically promoted the tumour growth of AGS (AGS‐KD versus AGS‐NC, *p* < 0.01) (Figure 2D). Similar results were obtained from the xenografted tumour of MKN28 cells in which RN181 expression was modulated (supplementary material, Figure S2D).

IHC staining of tumour sections confirmed the up‐regulation (AGS‐RN181 versus AGS‐RV, *p* < 0.01) and down‐regulation of RN181 (AGS‐KD versus AGS‐NC, *p* < 0.01) (Figure 2E) in GC tumours. Tumour cell proliferation in xenograft tumours was significantly decreased, as revealed by IHC for Ki67 (AGS‐RN181 versus AGS‐RV, *p* < 0.05), whereas tumour cell apoptosis was significantly increased, as demonstrated by anti‐activated caspase 3 staining (AGS‐RN181 versus AGS‐RV, *p* < 0.01). In agreement with the up‐regulation results, down‐regulation of RN181 increased tumour cell proliferation and decreased tumour cell apoptosis in xenograft tumours (AGS‐KD versus AGS‐NC, *p* < 0.05) and in AGS cells cultured *in vitro* (supplementary material, Figure S3). Taken together, the results suggest that RN181 functions as a tumour suppressor in the stomach to control the tumour growth of GC by decreasing tumour cell proliferation and increasing tumour cell apoptosis.

### RN181 controls cell cycle progression of GC

We performed cell cycle analyses by flow cytometry and western blots after synchronising the cells at the G1 phase by double‐thymidine block (supplementary material, Figure S4A,B). After release from the blockade, AGS‐RV cells progressed from the G1 to the S phase at 4–6 h, whereas AGS‐RN181 cells transited from the G1 to the S phase at 6–8 h (supplementary material, Figure S4A). Cell cycle analyses at the time point of 6 h (supplementary material, Figure S4C) showed 67.0% ± 5.6% of AGS‐RN181 cells in the G1 phase versus 13.0% ± 2.2% of AGS‐RV control cells in the G1 phase (*p* < 0.01). In contrast, 16.7% ± 3.0% in the S phase and 16.2% ± 1.8% in the G2 phase were observed for AGS‐RN181 versus 79.3% ± 6.6% in the S phase and 7.1% ± 1.8% in the G2 phase for AGS‐RV, respectively (both *p* < 0.01). Thus, the results indicate that RN181 controls the cell cycle transition from G1 to S phase and compels the GC cells to reside in the G1 phase for a prolonged period of 2 h.

Cell cycle analyses were also performed for AGS cells after knockdown of RN181 (supplementary material, Figure S4B). Compared with up‐regulation, down‐regulation of RN181 did not affect the G1–S phase transition but accelerated DNA synthesis, shortened the S phase, and promoted the cell cycle transition from S to G2 phase. After the release, AGS‐KD cells progressed from S to G2 phase at 8–10 h, whereas AGS‐NC cells transited from S to G2 phase at 10–12 h. Cell cycle distribution analyses at the time point of 10 h (supplementary material, Figure S4D) showed 20.7% ± 1.7% of AGS‐KD in S phase versus 61.4% ± 4.4% of AGS‐NC in S phase (*p* < 0.01). In contrast, 73.0%± 1.7% in G2 phase was observed for AGS‐KD compared with 32.8% ± 4.7% for AGS‐NC (*p* < 0.01). Thus, the results indicate that knockdown of RN181 expedites DNA synthesis, shortens the duration of the S phase, and promotes the S–G2 phase transition in GC cell cycles.

### RN181 regulates the G1–S transition by decreasing cyclin D1–CDK4 activity

**Figure 3:**
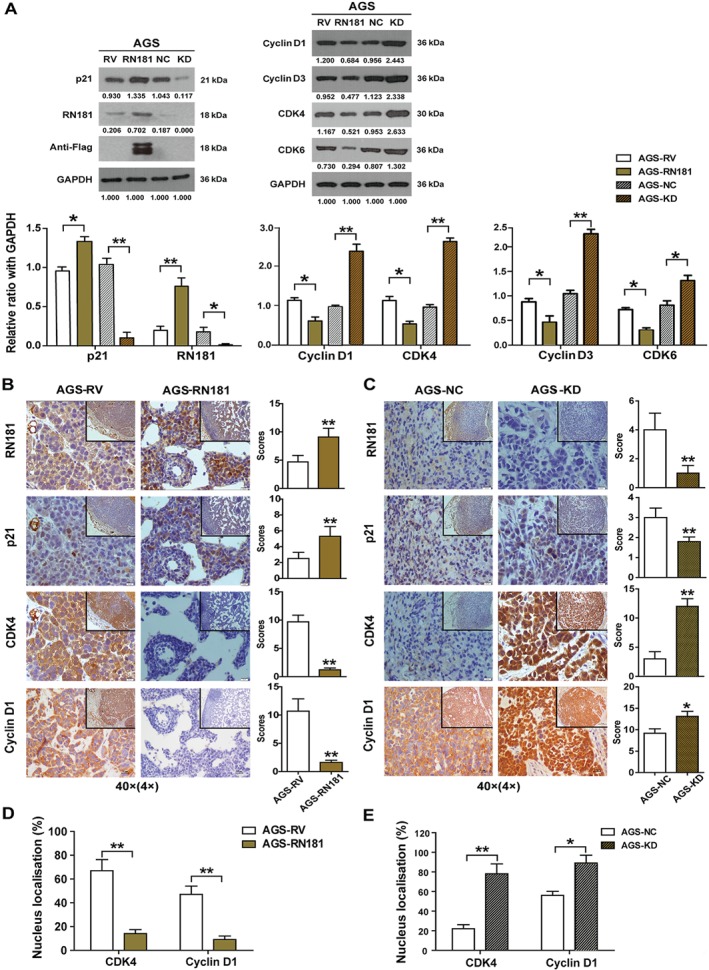
RN181 regulates the expression of G1/S checkpoint core components. (A) Western blotting analysis and densitometry quantification for the expression of RN181, p21, cyclin D1, cyclin D3, CDK4, and CDK6. The data (mean ± SD, *n* = 3) were analysed using Student's *t*‐test. *p < 0.05; **p < 0.01. (B) IHC staining and quantification for the expression of RN181, p21, cyclin D1, and CDK4 on tumour sections of AGS‐RN181 versus AGS‐RV cells. (C) IHC staining and quantification for the expression of RN181, p21, cyclin D1, and CDK4 on tumour sections of AGS‐KD versus AGS‐NC cells. The data (mean ± SD, *n* = 6) were analysed using Student's *t*‐test. *p < 0.05; **p < 0.01. Original magnification ×40 (inset ×10). (D) Up‐regulation of RN181 reduced the number of tumour cells that have nuclear staining for cyclin D1 and CDK4 in xenograft tumours of AGS‐RN181 versus AGS‐RV. (E) Down‐regulation of RN181 increased the number of tumour cells showing nuclear staining for cyclin D1 and CDK4 in xenograft tumours of AGS‐KD versus AGS‐NC. The data (mean ± SD, *n* = 6) were analysed using Student's *t*‐test. *p < 0.05; **p < 0.01.

We investigated the expression of G1/S checkpoint core proteins. Up‐regulation of RN181 increased the expression of p21 (AGS‐RN181 versus AGS‐RV, *p* < 0.05) but decreased the expression of cyclin D1, cyclin D3, CDK4, and CDK6 in AGS cells (AGS‐RN181 versus AGS‐RV, all *p* < 0.05) (Figure 3A). Consistently, down‐regulation of RN181 decreased the expression of p21 but increased the expression of cyclin D1, cyclin D3, CDK4, and CDK6 in AGS cells (AGS‐KD versus AGS‐NC, all *p* < 0.05). Similar results were obtained from MKN28 cells, in which knockdown of RN181 decreased the expression of p21 but increased the expression of cyclin D1 and CDK4 (supplementary material, Figure S5A).

To verify the *in vitro* results, we further investigated the correlation of RN181 expression with the activities of p21, cyclin D1, and CDK4 *in vivo*. IHC staining revealed that up‐regulation of RN181 significantly increased the expression of p21 but dramatically reduced the expression of cyclin D1 and CDK4 in xenograft tumours of AGS cells (AGS‐RN181 versus AGS‐RV, all *p* < 0.01) (Figure 3B). In agreement with up‐regulation, down‐regulation of RN181 significantly decreased the expression of p21 but increased the expression of cyclin D1 and CDK4 in xenograft tumours of AGS cells (AGS‐KD versus AGS‐NC control, all *p* < 0.05) (Figure 3C). Similar results were obtained from xenograft tumours of MKN28 cells in which RN181 was modulated (supplementary material, Figure S5B,C).

We also examined the cellular localisation of cyclin D1 and CDK4. Up‐regulation of RN181 dramatically reduced the percentage of tumour cells that have nuclear staining for cyclin D1 and CDK4 (AGS‐RN181 versus AGS‐RV, *p* < 0.01) (Figure 3D), while down‐regulation of RN181 significantly increased the percentage of tumour cells showing nuclear accumulation of cyclin D1 and CDK4 (AGS‐KD versus AGS‐NC, *p* < 0.05) (Figure 3E). Similar results were obtained from AGS cells cultured *in vitro* (supplementary material, Figure S6). Taken together, the results indicate that RN181 controls the G1–S phase transition by inhibiting the activity of cyclin D1–CDK4.

### Reconstitution of CDK4 rescues the inhibitory phenotype of GC cells by RN181

**Figure 4:**
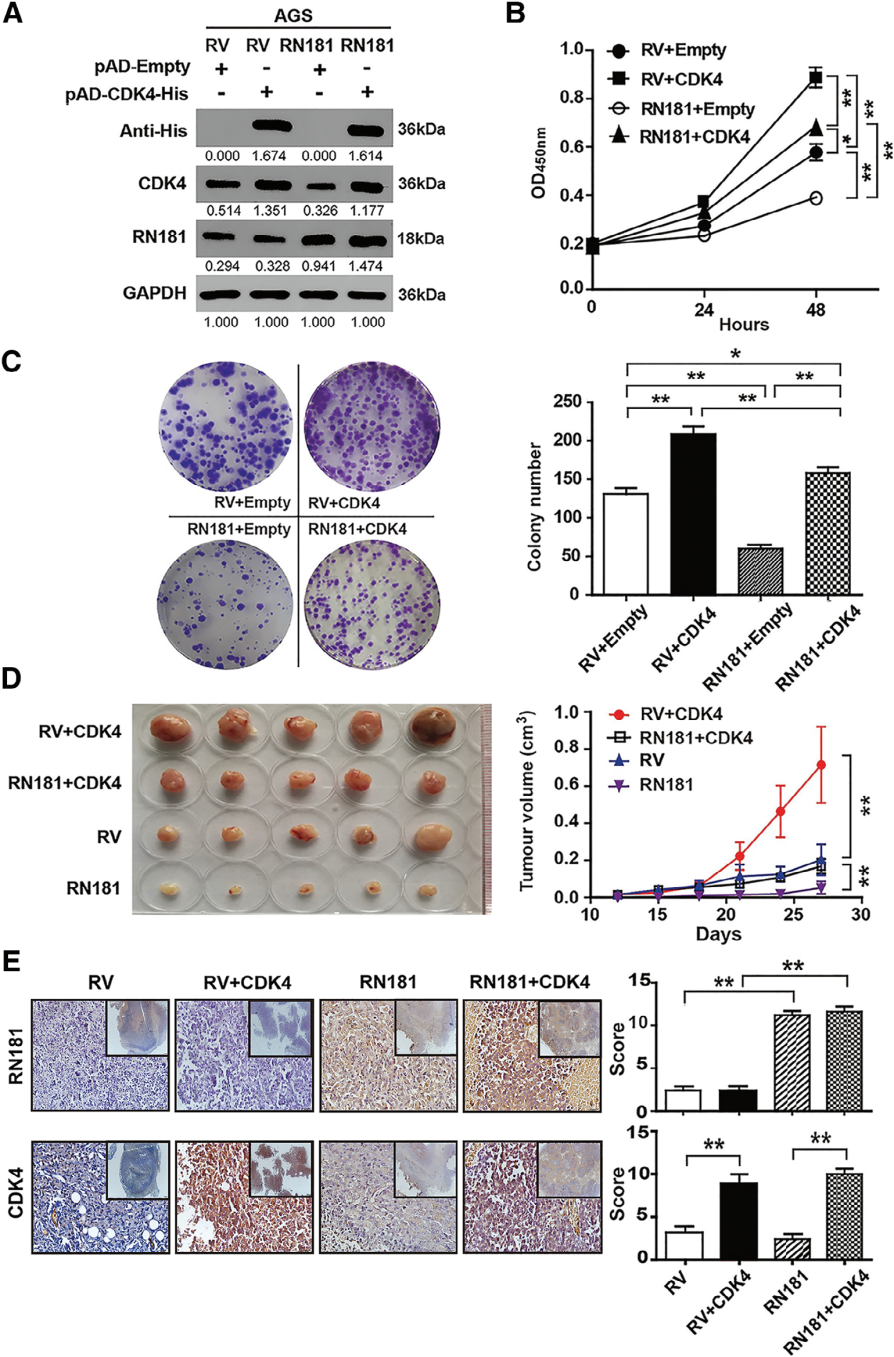
Reconstitution of CDK4 rescues the inhibitory phenotype of GC cells by RN181. (A) Western blotting showing the expression of RN181 and CDK4 in AGS cells. (B) Cell proliferation assays showed that reconstitution of CDK4 increased tumour cell growth of AGS at 24 and 48 h. Mean ± SD, *n* = 3. *p < 0.05; **p < 0.01. (C) Colony formation assays showed that reconstitution of CDK4 increased the colony numbers produced by AGS. Mean ± SD, *n* = 3. *p < 0.05; **p < 0.01. (D) Tumour growth of AGS cells in BALB/c nude mice. Mean ± SD, *n* = 6. **p < 0.01. (E) IHC staining and quantification for the expression of RN181 and CDK4 on tumour sections of AGS cells from panel D. Mean ± SD, *n* = 6. **p < 0.01. Original magnification ×40 (inset ×10).

We reconstituted the expression of CDK4 in AGS‐RN181 cells by adenovirus transductions. Western blots (Figure 4A) showed that transduction with pAD‐Empty adenovirus did not affect the expression of either RN181 (RV + Empty versus RV‐Empty; RN181 + Empty versus RN181‐Empty) or CDK4 (RV + Empty versus RN181 + Empty) in AGS cells, while transduction with pAD‐CDK4‐His increased the expression of CDK4 in both AGS‐RV control (RV‐CDK4 versus RV + CDK4) and AGS‐RN181 cells (RN181‐CDK4 versus RN181 + CDK4). Cell proliferation assays (Figure 4B) confirmed the inhibitory effect on AGS growth by RN181 (RN181 + Empty versus RV + Empty, *p* < 0.01). Interestingly, CDK4 reconstitution not only abolished the growth inhibition by RN181 (RN181 + CDK4 versus RN181 + Empty, *p* < 0.01) but also further increased the cell proliferation of AGS (RN181 + CDK4 versus RV + Empty, *p* < 0.05), highlighting a dominant role of CDK4 in control of the GC growth. However, the increased growth of AGS‐RV cells by CDK4 was much stronger than that of AGS‐RN181 (RV + CDK4 versus RN181 + CDK4, *p* < 0.01), suggesting that RN181 may also affect other molecules that regulate GC growth. Similar results were obtained from colony formation assays (Figure 4C).

We then implanted RN181‐ and CDK4‐overexpressing cells into nude mice (Figure 4D). Up‐regulation of RN181 significantly inhibited xenograft growth (RN181 versus RV, *p* < 0.01). Reconstitution of CDK4 eradicated the growth inhibition *in vivo* conferred by RN181 (RN181 + CDK4 versus RN181, *p* < 0.01). Consistent with the *in vitro* results, the promotion of AGS‐RV growth *in vivo* by CDK4 was significantly greater than that of AGS‐RN181 (RV + CDK4 versus RN181 + CDK4, *p* < 0.01). IHC staining of tumour sections confirmed the up‐regulation of RN181 (RN181 versus RV, *p* < 0.01) and CDK4 in both AGS‐RV tumour (RV + CDK4 versus RV, *p* < 0.01) and AGS‐RN181 tumour (RN181 + CDK4 versus RN181, *p* < 0.01) (Figure 4E).

### RN181 suppresses tumour growth by inhibition of ERK/MAPK in GC cells

**Figure 5:**
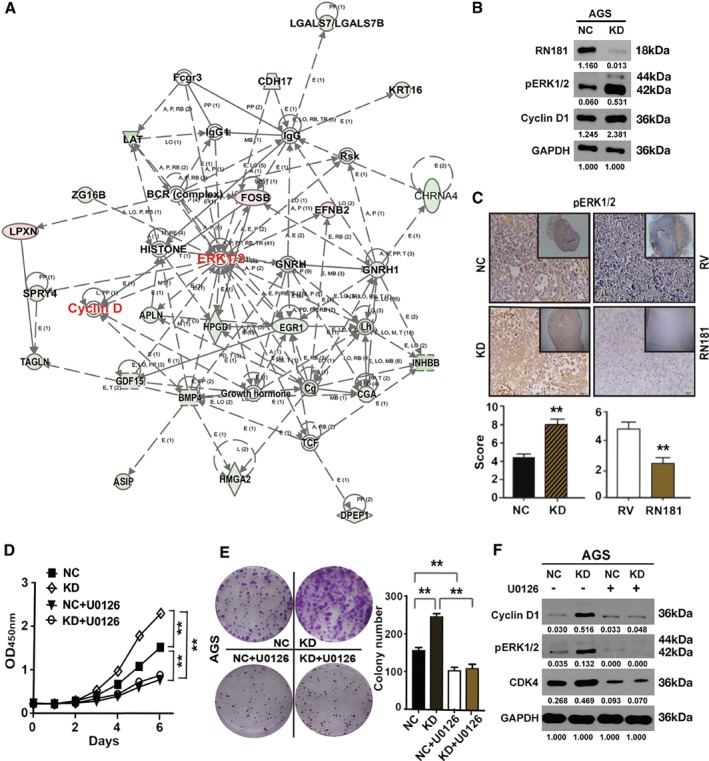
RN181 suppresses tumour growth by inhibition of ERK/MAPK signalling in AGS cells. (A) Ingenuity pathway analysis of differentially expressed genes regulated by RN181. (B) Western blotting showing up‐regulation of pERK1/2 and cyclin D1 by knockdown of RN181. (C) IHC staining and quantification for the expression of pERK1/2 on xenograft tumour sections of AGS cells. Mean ± SD, *n* = 6. **p < 0.01. Original magnification ×40 (inset ×10). (D) *In vitro* growth curves of AGS cells after knockdown of RN181 and treatment with the MEK1/2 inhibitor U0126. Mean ± SD, *n* = 3. **p < 0.01. (E) Colony formation assays showing colony numbers produced by AGS cells after knockdown of RN181 and treatment with U0125. Mean ± SD, *n* = 3. **p < 0.01. (F) Western blotting showing inhibition of the expression of pERK1/2, cyclin D1, and CDK4 in AGS cells by U0126.

We performed RNA‐Seq to compare gene expression profiles in AGS‐RN181 cells versus those in AGS‐RV cells. Ingenuity pathway analysis revealed that many differentially expressed genes regulated by RN181 were integrated in the pathways of ERK1/2–MAPK and cyclin D (Figure 5A), suggesting that RN181 may regulate ERK1/2–MAPK signalling which affects the cyclin D1–CDK4 activity that controls the cell cycle transition from G1 to S phase.

To address this hypothesis, we first evaluated the phosphorylation of pERK1/2 in GC cells. Down‐regulation of RN181 significantly increased the phosphorylation of pERK1/2 in AGS cells both cultured *in vitro* (KD versus NC) (Figure 5B) and xenografted *in vivo* (KD versus NC, *p* < 0.01) (Figure 5C). Consistent with the down‐regulation results, up‐regulation of RN181 dramatically decreased the phosphorylation of pERK1/2 (AGS‐RN181 versus AGS‐RV, *p* < 0.01). Similar results were observed in MKN28 cells in which RN181 was modulated (supplementary material, Figure S7A,B).

We then treated GC cells with U0126, a highly selective inhibitor of MEK1/MEK2. Cell proliferation (Figure 5D) and colony formation (Figure 5E) assays confirmed that down‐regulation of RN181 increased the abilities of cell growth and colony formation of AGS cells (AGS‐KD versus AGS‐NC, *p* < 0.01). Interestingly, U0126 treatment not only abolished the increased growth and colony formation abilities of AGS‐KD cells by down‐regulation of RN181 (AGS‐KD + U0126 versus AGS‐KD, *p* < 0.01) but also decreased the capacities of cell proliferation and colony formation of NC‐control cells (AGS‐NC + U0126 versus AGS‐NC, *p* < 0.01). Importantly, apart from inhibiting the phosphorylation of pERK1/2 in both AGS‐KD (KD + U0126 versus KD‐U0126) and AGS‐NC cells (NC + U0126 versus NC‐U0126), U0126 dramatically reduced the expression of cyclin D1 and CDK4 in both AGS‐KD (KD + U0126 versus KD‐U0126) and AGS‐NC cells (NC + U0126 versus NC‐U0126) (Figure 5F), suggesting that both cyclin D1 and CDK4 are downstream targets of ERK/MAPK signalling. Similar results were observed in MKN28 cells in which RN181 was modulated (supplementary material, Figure S7C–E). Taken together, the results indicate that RN181 suppresses the tumour growth of GC by inhibition of the ERK/MAPK pathway and consequently control of the activity of cyclin D1–CDK4 that regulates the cell cycle transition from G1 to S phase.

### Correlation between RN181 and cyclin D1/CDK4 in GC clinical samples

**Figure 6:**
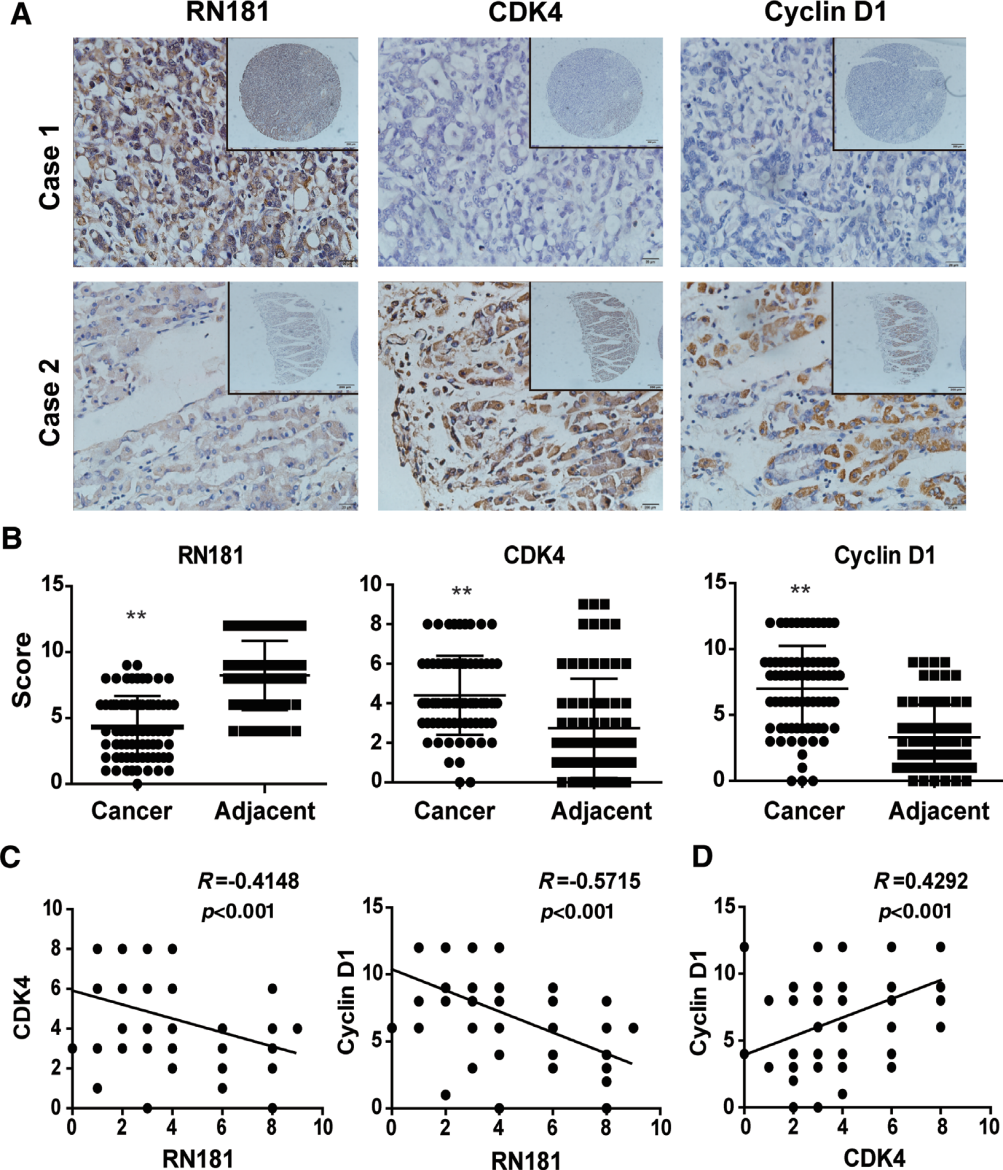
Expression and correlation analyses of RN181, cyclin D1, and CDK4 in GC specimens. (A) Representative images of RN181, cyclin D1, and CDK4 expressed in GC tumour tissue and adjacent non‐tumour tissue examined by IHC. (B) Quantification of IHC scoring for the expression of RN181, cyclin D1, and CDK4 in GC tumour tissue and adjacent non‐tumour tissue. The data (mean ± SE, *n* = 75) were analysed using sample‐paired *t*‐test. **p < 0.01. (C) Pearson correlation analysis showing reverse correlations of RN181 expression with the expression of cyclin D1 and CDK4 in GC tumours, respectively. (D) Pearson correlation analysis showing the positive correlation between the expression of cyclin D1 and CDK4 in GC tumours.

We further investigated the expression of RN181, cyclin D1, and CDK4 in GC clinical specimens by IHC (Figure 6A). Quantitative analysis by scoring the staining showed that the expression of RN181 was significantly down‐regulated in tumour tissues versus adjacent non‐tumour tissues, while the expression of cyclin D1 and CDK4 was significantly up‐regulated in tumour tissues versus adjacent tissues, respectively (all *p* < 0.001) (Figure 6B). Pearson correlation analyses revealed that the expression of RN181 was reversely associated with the levels of cyclin D1 and CDK4, respectively (all *p* < 0.001) (Figure 6C). In contrast, the expression of cyclin D1 was positively correlated with the expression of CDK4 (*p* < 0.001) (Figure 6D). Altogether, the results substantiate the role of the RN181–cyclin D1/CDK4 pathway in control of the tumour development of GC.

## Discussion

The activation of oncogenes and inactivation of tumour suppressor genes play major roles in the pathogenesis of GC 14, 15. Recently, several novel putative tumour suppressors such as BCL6B 16, CPEB1 17, ZNF331 18, ZNF545 19 and CHIP 20 have been identified in GC. In this study, we demonstrated that RN181 was significantly down‐regulated in tumour tissues versus adjacent non‐tumour tissues. Remarkably, RN181 expression was inversely associated with tumour differentiation, tumour size, clinical stage, and patient's overall survival. These results suggest that RN181 may be involved in the pathogenesis and development of GC and may serve as a biomarker for predicting the outcome of GC patients. To study the possibility that RN181 could affect the carcinogenesis and development of GC, we modulated the expression of RN181 in GC cells. We found that up‐regulation of RN181 significantly inhibited tumour growth, while down‐regulation of RN181 promoted tumour growth by regulation of tumour cell proliferation and apoptosis *in vitro* and *in vivo*. Therefore, we conclude that RN181 is a novel tumour suppressor in the stomach and controls the tumour growth of GC.

Many tumour suppressors constrain cell growth and proliferation by affecting a variety of signalling pathways that impinge on the core cell‐cycle machinery 14, 21. To explore underlying cellular mechanisms, we performed cell cycle analyses. Up‐regulation of RN181 compelled GC cells to reside in the G1 phase for an extra 2 h, whereas down‐regulation of RN181 accelerated DNA synthesis and increased the cell cycle progression from the S to the G2 phase for 2 h. Thus, we conclude that suppression of tumour cell proliferation by RN181 is attributed to the delay of the cell cycle transition from the G1 to the S phase of GC. Deregulation of signalling networks during the G1 phase of the cell cycle represents major driving forces in the tumourigenesis and development of cancer 21. Many oncogenes promote cell proliferation by accelerating the G1–S phase transition. Cyclin D1, CDK4, and CDK6 are among the core players for such G1–S phase transition 21, 22, 23, 24. We found that up‐regulation of RN181 significantly decreased the expression of cyclin D1 and CDK4, while down‐regulation of RN181 increased the expression of cyclin D1 and CDK4 in GC cells. Strikingly, knockdown of RN181 significantly increased nuclear localisation of cyclin D1 and CDK4 in tumour cells, suggesting an increase of cyclin D1–CDK4 activity 22, 25, 26. Interestingly, CDK4‐deficient MEF cells were reported to stay in the G1 phase for a prolonged period 27, suggesting that postponement of the G1–S phase transition may be mediated by decreasing CDK4 expression by RN181. To corroborate the role of CDK4 in the inhibition of tumour growth by RN181, we reconstituted the expression of CDK4 in RN181‐overexpressing GC cells. Indeed, CDK4 reconstitution not only rescued the growth inhibitory phenotype conferred by RN181 both *in vitro* and *in vivo* but also further increased the cell growth and colony formation of GC, highlighting a dominant role of CDK4 in the control of GC growth by RN181.

To further investigate underlying mechanisms, we profiled gene expression by RNA‐Seq. We found that many differentially expressed genes regulated by RN181 were tightly integrated in the pathways of ERK/MAPK and cyclin D1. In many cell types, both transcription of cyclin D1 and CDK4 and assembly of cyclin D1 with CDK4 rely on activation of RAS–RAF–MEK–ERK signalling 28. In GC, several factors including the RAF/MEK/ERK pathway 29 are involved in cyclin D1 up‐regulation. We previously found that RN181 could inhibit the ERK/MAPK pathway in hepatocellular carcinoma 12. Therefore, we hypothesised that the suppression of tumour growth of GC by RN181 may undergo by reducing the expression and assembly of cyclin D1 with CDK4 mediated by inhibition of ERK/MAPK signalling in the stomach. Indeed, up‐regulation of RN181 significantly inhibited the phosphorylation of pERK1/2 and down‐regulation of RN181 greatly increased the phosphorylation of pERK1/2 in GC cells *in vitro* and *in vivo*. Remarkably, inhibition of MEK/ERK/MAPK signalling by U0126 not only eliminated the increased growth of tumour cells by down‐regulation of RN181 but also equally decreased the abilities of proliferation and colony formation of GC cells. More importantly, U0126 dramatically reduced the expression of cyclin D1 and CDK4, implying that both cyclin D1 and CDK4 are downstream targets of ERK/MAPK signalling in GC. Tob1, which is known to repress the expression of cyclin D1 and CDK4 in GC 30, is also phosphorylated by ERK/MAPK kinases, which relieves the transcriptional repression of cyclin D1 31. ASK1, which was known to participate in colon and skin tumourigenesis, could increase the expression of cyclin D1 through AP‐1 activation and cyclin D1 could up‐regulate ASK1 via the Rb‐E2F pathway in GC to stimulate tumour growth 32. Thus, either Tob1 or ASK1 may also be involved in the suppression of tumour growth of GC by RN181.

To verify the results from *in vitro* and *in vivo* models, we further investigated the activity of cyclin D1 and CDK4 in another cohort of GC clinical specimens. We found that the expression of cyclin D1 and CDK4 was significantly elevated in GC tissues versus adjacent non‐tumour tissues. Indeed, up‐regulation of cyclin D1 and CDK4 was observed in a wide spectrum of tumours 22, 24, 26. Remarkably, about 37–56% of GC patients expressed abnormally high levels of cyclin D1 32, 33, 34. More importantly, the expression of RN181 was reversely correlated with the expression of cyclin D1 and CDK4, respectively, while the expression level of cyclin D1 was positively associated with the expression of CDK4, highlighting the role of the RN181–cyclin D1/CDK4 pathway in the tumour development of GC. Accordingly, we speculate that specific inhibition of cyclin D1/CDK4 activity may produce clinical benefits for GC patients who have elevated expression of cyclin D1 and CDK4 in their tumours. Importantly, palbociclib, a small‐molecule inhibitor of CDK4 and CDK6, combined with letrozole has recently been approved to treat postmenopausal women with ER‐positive, HER2‐negative advanced breast cancer as a first‐line therapy 35.

In conclusion, we have demonstrated that RN181 is down‐regulated in GC and is inversely associated with some clinicopathological features and patient's overall survival. RN181 functions as a tumour suppressor in the stomach to inhibit tumour growth *in vitro* and *in vivo* by control of the cell cycle progression from G1 to S phase. Mechanistically, RN181 inhibited ERK/MAPK signalling, thereby regulating cyclin D1–CDK4 activity and consequently controlling the cell cycle progression. Reconstitution of CDK4 rescued the inhibitory phenotype conferred by RN181. Importantly, in clinical tumour samples, RN181 was reversely correlated with the expression of cyclin D1 and CDK4, highlighting the role of the RN181–cyclin D1/CDK4 pathway in the control of tumour growth. Our results provide new insights into the carcinogenesis and development of GC and facilitate the development of novel intervention strategies against GC by disrupting the ERK/MAPK–cyclin D1/CDK4 pathway. RN181 may become a prognostic biomarker for predicting the outcome of GC patients.

## Author contributions statement

SW, XW, YG, and J‐LL designed the research. SW, XW, YG, YP, ND, QX, and XZ performed experiments. SW, XW, YG, and J‐LL analysed data. YW, ML, and J‐LL supervised the research. J‐LL wrote the paper. All the authors approved the manuscript.

**SUPPLEMENTARY MATERIAL ONLINE**

**Supplementary materials and methods**

**Supplementary figure legends**

**Figure S1.** Representative images of RN181 expressed in GC tumour and adjacent non‐tumour tissues

**Figure S2.** Alterations of RN181 expression regulate the tumour growth of MKN28 and MKN45 cells

**Figure S3.** Alternation of RN181 expression affects the apoptosis of AGS cells

**Figure S4.** RN181 regulates cell cycle progression of GC

**Figure S5.** RN181 regulates the expression of G1/S checkpoint core components in MKN28 cells

**Figure S6.** RN181 knockdown increases translocation of cyclin D1 and CDK4 from the cytoplasm to the nucleus of AGS cells

**Figure S7.** RN181 suppresses tumour growth by inhibition of ERK/MAPK signalling in MKN28 cells

**Table S1.** Association of the expression of RN181 with clinical features of 165 patients with gastric cancer according to the expression of RN181 in tumour tissues

## Supporting information


**Supplementary materials and methods**

**Supplementary figure legends**

**Table S1.** Association of the expression of RN181 with clinical features of 165 patients with gastric cancer according to the expression of RN181 in tumour tissues

**Figure S1.** Representative images of RN181 expressed in GC tumour and adjacent non‐tumour tissues.

**Figure S2.** Alterations of RN181 expression regulate the tumour growth of MKN28 and MKN45 cells.

**Figure S3.** Alternation of RN181 expression affects the apoptosis of AGS cells.

**Figure S4.** RN181 regulates cell cycle progression of GC.

**Figure S5.** RN181 regulates the expression of G1/S checkpoint core components in MKN28 cells.

**Figure S6.** RN181 knockdown increases translocation of cyclin D1 and CDK4 from the cytoplasm to the nuclei of AGS cells.

**Figure S7.** RN181 suppresses tumour growth by inhibition of ERK/MAPK signalling in MKN28 cells.
