# Supplementary material for: RN181 is a tumour suppressor in gastric cancer by regulation of the ERK/MAPK–cyclin D1/CDK4 pathway
Source: J Pathol. 2019 Apr 11;248(2):204–16. doi: 10.1002/path.5246 (PMC6593865; doi:10.1002/path.5246)
Supplement: Supplementary file 1 — Supplementary materials and methods [file PATH-248-204-s001.docx]

**RN181 is a tumour suppressor in gastric cancer by regulation of the ERK/MAPK–cyclin D1/CDK4 pathway**

Wang S *et al*. *J Pathol* DOI: 10.1002/path.5246

**Supplementary materials and methods**

Reference numbers refer to the main text reference list

**Vector construction and transfection**

Construction of the pLNCX2/RN181 vector, lentivirus packaging in GP2-293, and virus transduction were performed as described previously [12]. The lentiviruses, packaged with the pGLV-U6-GFP vector containing either shRNA sequence against RN181 (5'-GGCGTCCTATTTCGATGAACA-3') or control sequence (5'-GTTCTCCGAACGTGTCACG-3'), were purchased from Shanghai GenePharma Co, Ltd (PR China) and transduced into GC cell lines according to the manufacturer’s instructions. GFP-positive cells for either up-regulation of RN181 or down-regulation of RN181 were sorted out by flow cytometry.

Pre-made pAD-CDK4 adenoviruses with human CDK4 (NM_000075) fused with C-terminal Flag and His tag at its C-terminus driven by the CMV promoter [VH864934; Vigene Biosciences Inc (https://[www.vigenebio.cn](http://www.vigenebio.cn))] were transduced into AGS cells according to the manufacturer’s instructions.

**RT-qPCR**

RNA extractions, cDNA synthesis, and qPCR were carried out as described previously [12]. The experiment was repeated at least three times.

**Western blotting**

Protein extractions and western blotting were performed as described previously [12]. Antibodies contained in Cell Cycle Regulation Samper Kit 1/2 (Cell Signaling, Danvers, MA. USA) were used for western blotting. Protein bands on the blots were visualised by autoradiography and quantified by densitometry analyses using a Versadoc Imaging System model 3000. The experiment was repeated at least three times.

**RNA-deep sequencing (RNA-Seq)**

RNA-Seq was performed and analysed by BGI Group, Shenzhen, PR China. In brief, mRNAs were enriched from the RNA extracts of AGS cells, fragmented with divalent cations, and converted to single-strand cDNA. After converting the overhangs into blunt ends, sequencing adapters were ligated to the end of cDNA. The fragments were then enriched by PCR. The PCR products were then sequenced with Illumina HisSeq.

**Cell proliferation assays**

Cell proliferation assays were performed using Cell Counting Kit-8 (CCK-8; Sigma-Aldrich, St Louis, MO, USA) according to the manufacturer’s instructions. In brief, GC cells (5000 cells per well/500 μl) plus or minus U0126 (20 μm) (#9903, Cell Signaling) were seeded in quadruplicate in 24-well plates and cultured at 37℃ with 5% CO_2_ for 1–6 days. Forty microlitres of CCK-8 solution was added to each well and incubated for another 4 h. Absorbance at 450 nm was measured with a background subtraction. A calibration curve was prepared using the data obtained from wells that contained known numbers of viable cells. The experiment was repeated at least three times.

**Colony formation assays**

Colony formation assays were performed with six-well plates (500 cells per well) plus or minus U0126 (20 μm) as described previously [12]. After incubation at 37°C with 5% CO_2_ for 15–20 days, during which the supernatants were replaced with fresh medium every 2 days, cell colonies formed in the plate were stained with crystal violet after being fixed by methanol and then counted. The average number of colonies was calculated from four replicates. The experiment was repeated at least three times.

**Cell cycle analyses**

Synchronisation of cell cycles was achieved by double-thymidine blockade at the G1 phase. Cell suspensions (2 × 10^4^ cells/2 ml per well) were plated in triplicate in six-well plates. Thymidine (Sigma-Aldrich) was added to a final concentration of 4 mm after the cells adhered on the plate (about 6–8 h). The tumour cells were then incubated at 37°C for 16 h and the supernatants were replaced with an equal amount of fresh medium without adding thymidine. After being cultured for 10 h, 4 mm thymidine was added to the culture again for another 16 h. The cell cycles were then released from the G1 phase after the double-thymidine blockade by replacing the supernatant with normal fresh medium. The synchronised cells were collected every 2 h either for flow cytometry analysis or for protein extraction for western blotting. The collected cells were washed twice with PBS and fixed with chilled 70% alcohol at −20°C for 24 h. The cells were centrifuged at 1000 rpm for 5 min and washed twice with cold PBS. After being treated with 20 μl of RNase A (20 μg/ml) at 37°C for 30 min, the cells were stained with 25 μg/ml propidium iodide (Sigma-Aldrich) at room temperature for 30 min. Cell cycle distribution was then carried out by flow cytometry.

**Immunofluorescence assays**

Cells were cultured on glass coverslip in six-well plates at 37°C with 5% CO_2_ for 12 h and fixed with 4% paraformaldehyde for 30 min. Cells were then washed three times with PBS and permeabilised in PBS containing 0.25% Triton X-100 (Biyuntian, PR China) for 15 min. Cells were then blocked with 10% goat serum at room temperature for 30 min, followed by incubation with the primary antibody against cyclin D1 or CDK4 in a moist chamber at 4°C overnight. After three washes, cells were incubated with an Alexa Fluor 488 conjugated secondary antibody at room temperature for 1 h. Cell nuclei were stained with DAPI (Biyuntian). Each coverslip was then mounted onto a slide and fluorescence images were taken using a Zeiss LSM880 confocal microscope (Carl Zeiss, Oberkochen, Germany). The integral optical density (IOD) of protein expression was calculated automatically by Image-Pro Plus software (version 6.0; Media Cybernetics, Bethesda, MD, USA).
