# Supplementary material for: RN181 is a tumour suppressor in gastric cancer by regulation of the ERK/MAPK–cyclin D1/CDK4 pathway
Source: J Pathol. 2019 Apr 11;248(2):204–16. doi: 10.1002/path.5246 (PMC6593865; doi:10.1002/path.5246)
Supplement: Supplementary file 2 — Supplementary figure legends [file PATH-248-204-s010.docx]

**RN181 is a tumour suppressor in gastric cancer by regulation of the ERK/MAPK–cyclin D1/CDK4 pathway**

Wang S *et al*. *J Pathol* DOI: 10.1002/path.5246

**Supplementary figure legends**

**Figure S1. Representative images of RN181 expressed in GC tumour and adjacent non-tumour tissues.** The intensity of RN181 immunostaining was scored as negative (0+), low (1+), medium (2+) or high (3+) in gastric tissues. Adjacent non-tumour tissues showed high expression (3+), whereas GC tissues showed medium (2+), low (1+) or undetectable expression (0+) of RN181. Original magnification: ×4 and ×40.

**Figure S2. Alterations of RN181 expression regulate the tumour growth of MKN28 and MKN45 cells.** (A) Lentivirus-mediated transduction to knock down the expression of RN181 in GC cells at mRNA and protein levels as determined by QRT-PCR (left panel) and western blotting (right panel), respectively. The data (mean ± SD, *n* = 3) were analysed using Student’s *t*-test. ***p* < 0.01. (B) Cell proliferation assays showed that down-regulation of RN181 increased the growth of MKN28 cells (left panel) and MKN45 cells (right panel) *in vitro*. The data (mean ± SD, *n* = 6) were analysed using Student’s *t*-test. **p* < 0.05; ***p* < 0.01. (C) Colony formation assays showed that down-regulation of RN181 increased the number of colonies formed by MKN28 and MKN45 cells *in vitro*. The data (mean ± SD, *n* = 6) were analysed using Student’s *t*-test. ***p* < 0.01. (D) Down-regulation of RN181 increased tumour growth (top panels), while up-regulation of RN181 decreased tumour growth (bottom panels) of MKN28 cells in BALB/c nude mice. The data (mean ± SD, *n* = 5) were analysed using sample-paired *t*-test. ***p* < 0.01.

**Figure S3. Alternation of RN181 expression affects the apoptosis of AGS cells.** (A) Western blotting showing that up-regulation of RN181 increased the apoptosis, while down-regulation of RN181 decreased the apoptosis of AGS cells. (B) Flow cytometry assay showing that up-regulation of RN181 increased the apoptosis, while down-regulation of RN181 decreased the apoptosis of AGS cells. The data (mean ± SD, *n* = 4) were analysed using sample-paired *t*-test. **p* < 0.05; ***p* < 0.01.

**Figure S4. RN181 regulates cell cycle progression of GC.** (A) Up-regulation of RN181 in AGS cells resulted in arrest of cell cycle progression from G1 to S phase as shown by flow cytometry analysis of propidium iodide incorporation and by western blotting. (B) Down-regulation of RN181 in AGS cells led to acceleration of DNA synthesis, shortened the duration of the S phase, and promoted the S–G2 phase transition in the cell cycle. (C) Cell cycle distribution analysis of RN181-up-regulating cells at the time point of 6 h after release from the blockade as indicated by an arrowhead in panel A. (D) Cell cycle distribution analysis of RN181-knockdown cells at the time point of 10 h after release from the blockade as indicated by an arrowhead in panel B. The data (mean ± SD, *n* = 3) were analysed using Student’s *t*-test. ***p* < 0.01.

**Figure S5. RN181 regulates the expression of G1/S checkpoint core components in MKN28 cells.** (A) Western blotting analysis and densitometry quantification for the expression of cyclin D1, CDK4, p21, and RN181. The data (mean ± SD, *n* = 3) were analysed using Student’s *t*-test. **p* < 0.05. (B) IHC and quantification for the expression of cyclin D1, CDK4, and RN181 in xenograft tumours of MKN28-RN181 versus MKN28-RV control. The data (mean ± SD, *n* = 6) were analysed using Student’s *t*-test. ***p* < 0.01. (C) IHC and quantification for the expression of cyclin D1, CDK4, and RN181 in MKN28 xenograft tumours of MKN28-KD versus MKN28-NC control. The data (mean ± SD, *n* = 6) were analysed using Student’s *t*-test. ***p* < 0.01.

**Figure S6. RN181 knockdown increases translocation of cyclin D1 and CDK4 from the cytoplasm to the nuclei of AGS cells.** (A) Immunofluorescent assays showing that RN181 knockdown increased the expression of cyclin D1 and CDK4 in AGS nuclei. IOD: integral optical density. The data (mean ± SD, *n* = 4 fields) were analysed using Student’s *t*-test. ***p* < 0.01. (B) Western blotting analysis showing that RN181 knockdown decreased the expression of cyclin D1 and CDK4 in cytoplasm, while it increased the expression of cyclin D1 and CDK4 in the nuclei of AGS cells. GAPDH: cytoplasmic protein loading control; Histone H3: nuclear protein loading control.

**Figure S7. RN181 suppresses tumour growth by inhibition of ERK/MAPK signalling in MKN28 cells.** (A) Western blotting showing that down-regulation of RN181 increased the phosphorylation of pERK1/2 and cyclin D1 expression in MKN28 cells. (B) IHC and quantification showing that down-regulation of RN1181 increased the phosphorylation of pERK1/2, while up-regulation of RN181 decreased the level of pERK1/2 in xenograft tumours of MKN28 cells. Mean ± SD, *n* = 6. ***p* < 0.01. (C) Cell proliferation assays showing that RN181 knockdown increased the tumour growth of MKN28 cells that was completely abolished by U0126 treatment. Mean ± SD, *n* = 3. ***p* < 0.01. (D) Colony formation assays showing that RN181 knockdown increased the colony formation of MKN28 cells that was completely eliminated by U0126 treatment. Mean ± SD, *n* = 3. ***p* < 0.01. (E) Western blotting showing up-regulation of pERK1/2, cyclin D1, and CDK4 by knockdown of RN181. Treatment with U0126 not only decreased the phosphorylation of pERK1/2 but also decreased the expression of cyclin D1 and CDK4 in MKN28 cells.
