# Supplementary material for: RN181 is a tumour suppressor in gastric cancer by regulation of the ERK/MAPK–cyclin D1/CDK4 pathway
Source: J Pathol. 2019 Apr 11;248(2):204–16. doi: 10.1002/path.5246 (PMC6593865; doi:10.1002/path.5246)
Supplement: Supplementary file 3 — Table S1. Association of the expression of RN181 with clinical features of 165 patients with gastric cancer according to the expression of RN181 in tumour tissues [file PATH-248-204-s002.docx]

**RN181 is a tumour suppressor in gastric cancer by regulation of the ERK/MAPK–cyclin D1/CDK4 pathway**

Wang S *et al*. *J Pathol* DOI: 10.1002/path.5246

**Table S1.** Association of the expression of RN181 with clinical features of 165 patients with gastric cancer according to the expression of RN181 in tumour tissues

|  | ***N*** | **RN181 expression** | | **Mean rank** | | **Statistics** | *p* |
| --- | --- | --- | --- | --- | --- | --- | --- |
|  |  | **Low (≤ 3)** | **High (> 3)** |  |  |  |  |
| Gender | | | | | | −1.548 | 0.122 |
| Male | 118 | 98 | 20 | | 80.48 |  |  |
| Female | 47 | 34 | 13 | | 89.32 |  |  |
| Age, years | | | | | | 1.362 | 0.173 |
| ≤ 50 | 15 | 14 | 1 | | 72.00 |  |  |
| 51–70 | 88 | 71 | 17 | | 82.44 |  |  |
| ≥ 71 | 62 | 47 | 15 | | 86.46 |  |  |
| Differentiated | | | | | | −2.045 | 0.041 |
| Well | 45 | 32 | 13 | | 90.33 |  |  |
| Moderately | 112 | 92 | 20 | | 81.23 |  |  |
| Poorly | 8 | 8 | 0 | | 66.50 |  |  |
| Clinical stage | | | | | | −2.765 | 0.006 |
| Ⅰ | 15 | 10 | 5 | | 94.00 |  |  |
| Ⅱ | 49 | 35 | 14 | | 90.07 |  |  |
| Ⅲ | 89 | 75 | 14 | | 79.48 |  |  |
| Ⅳ | 12 | 12 | 0 | | 66.50 |  |  |
| Tumour size, cm | | | | | | −2.484 | 0.013 |
| ≤ 2.5 | 44 | 31 | 13 | | 88.78 |  |  |
| 2.5 < *d* < 6.5 | 100 | 81 | 19 | | 80.30 |  |  |
| ≥ 6.5 | 17 | 17 | 0 | | 65.00 |  |  |
| Survival time, years | | | | | | −2.915 | 0.004 |
| < 5 | 106 | 92 | 14 | | 77.40 |  |  |
| ≥ 5 | 59 | 40 | 19 | | 93.07 |  |  |
