# Supplementary figures and images for: RN181 is a tumour suppressor in gastric cancer by regulation of the ERK/MAPK–cyclin D1/CDK4 pathway
Source: J Pathol. 2019 Apr 11;248(2):204–16. doi: 10.1002/path.5246 (PMC6593865; doi:10.1002/path.5246)

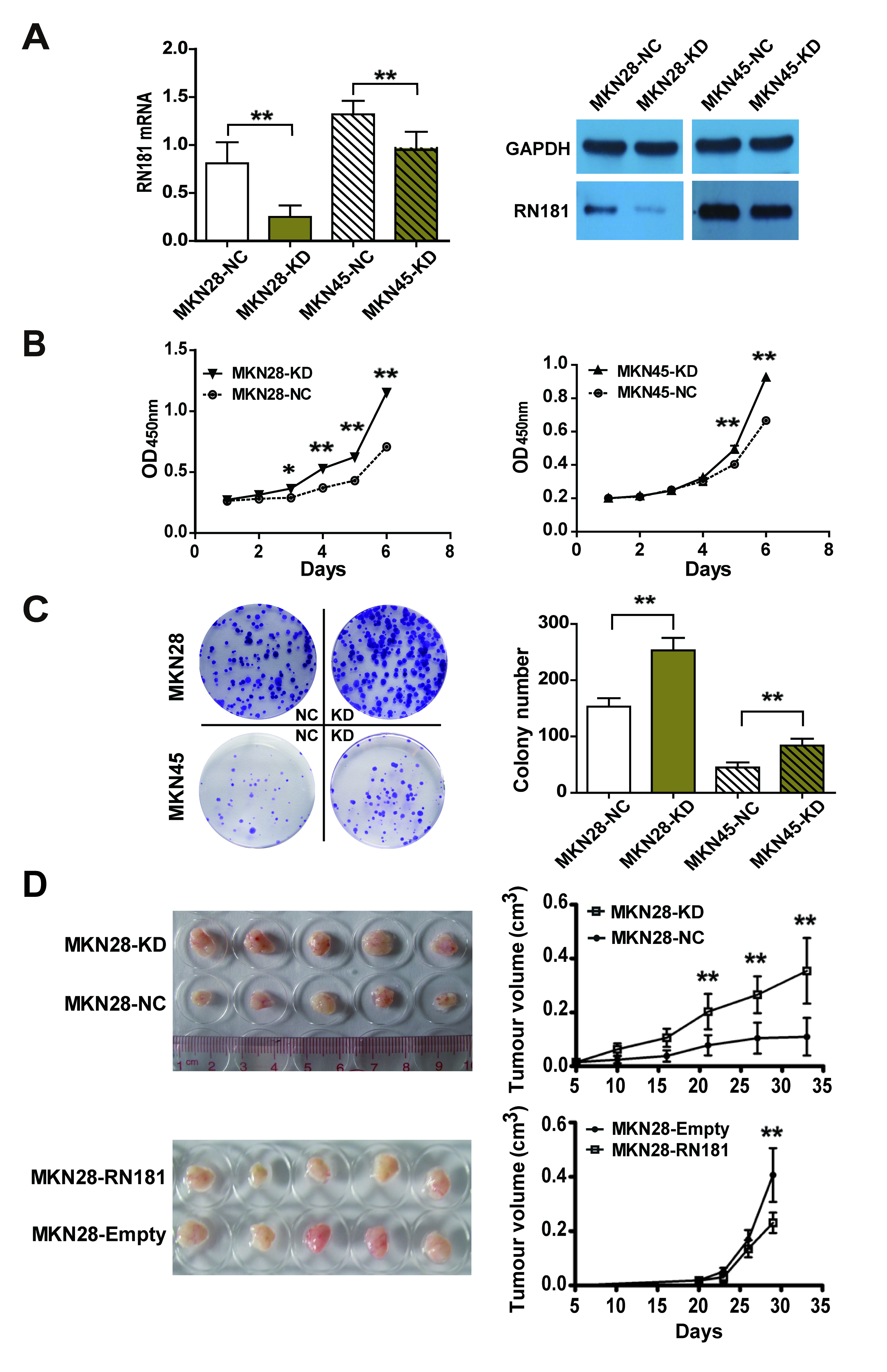

Supplement: Supplementary file 5 — Figure S2. Alterations of RN181 expression regulate the tumour growth of MKN28 and MKN45 cells. [file PATH-248-204-s004.tif]

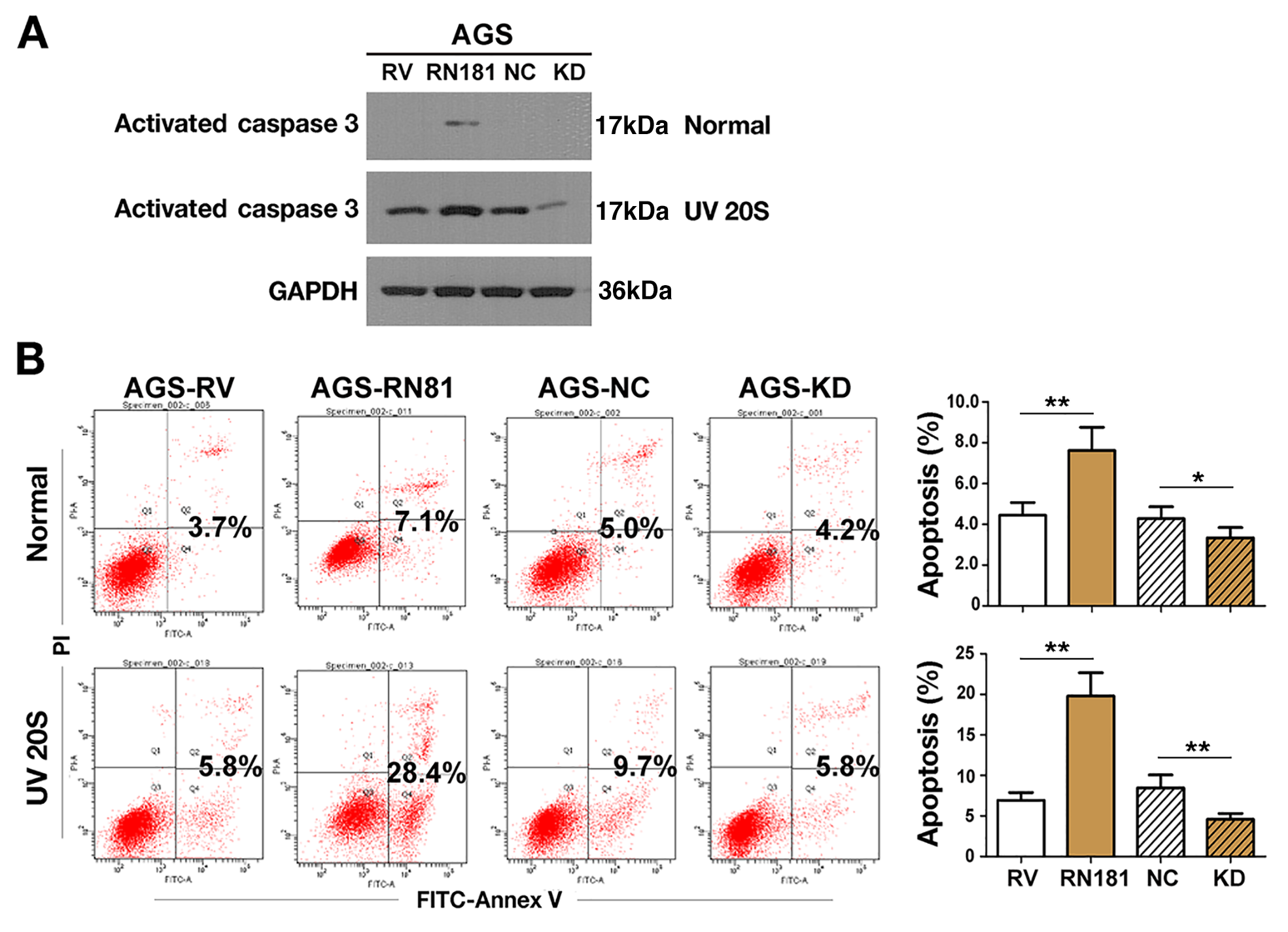

Supplement: Supplementary file 6 — Figure S3. Alternation of RN181 expression affects the apoptosis of AGS cells. [file PATH-248-204-s005.tif]

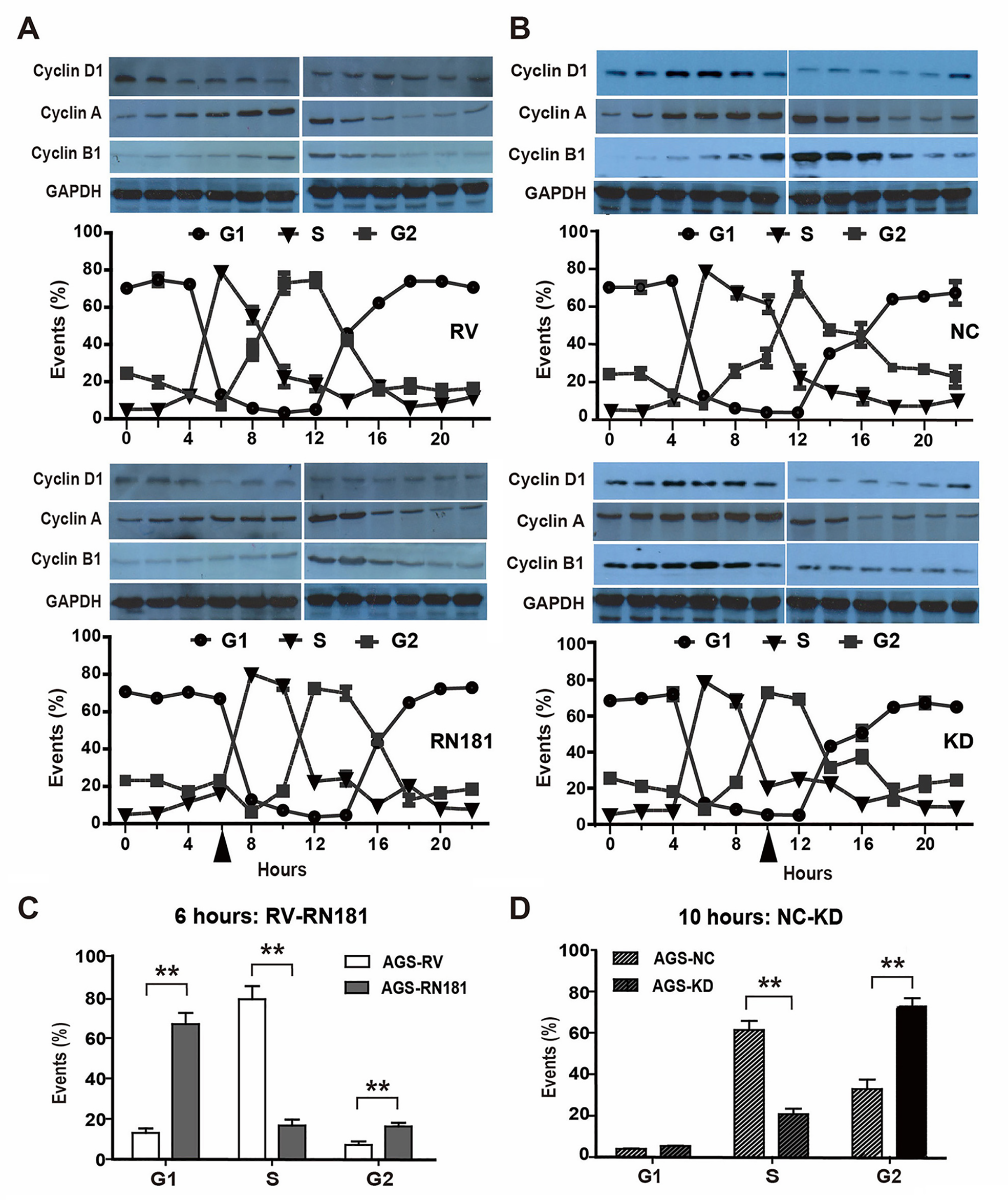

Supplement: Supplementary file 7 — Figure S4. RN181 regulates cell cycle progression of GC. [file PATH-248-204-s006.tif]

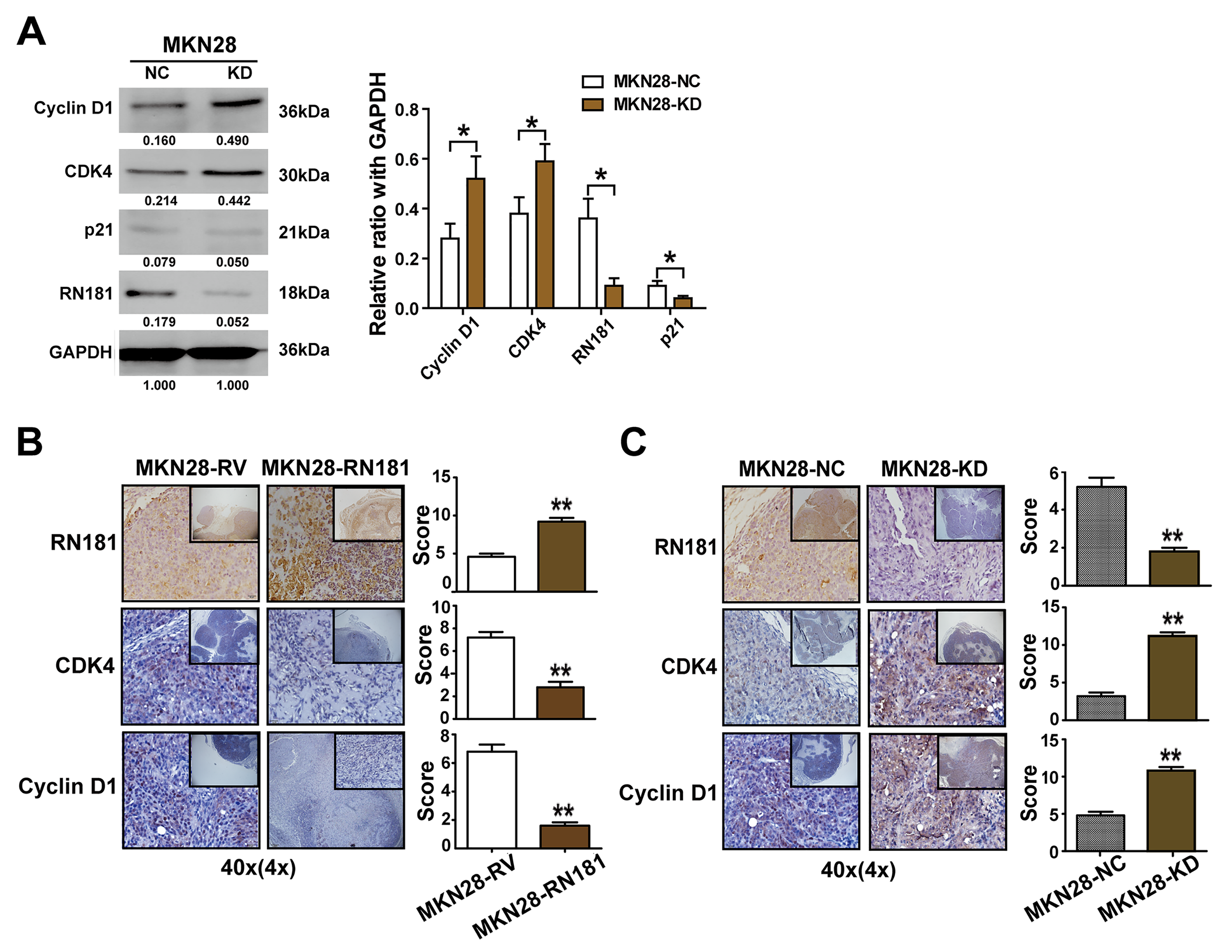

Supplement: Supplementary file 8 — Figure S5. RN181 regulates the expression of G1/S checkpoint core components in MKN28 cells. [file PATH-248-204-s007.tif]

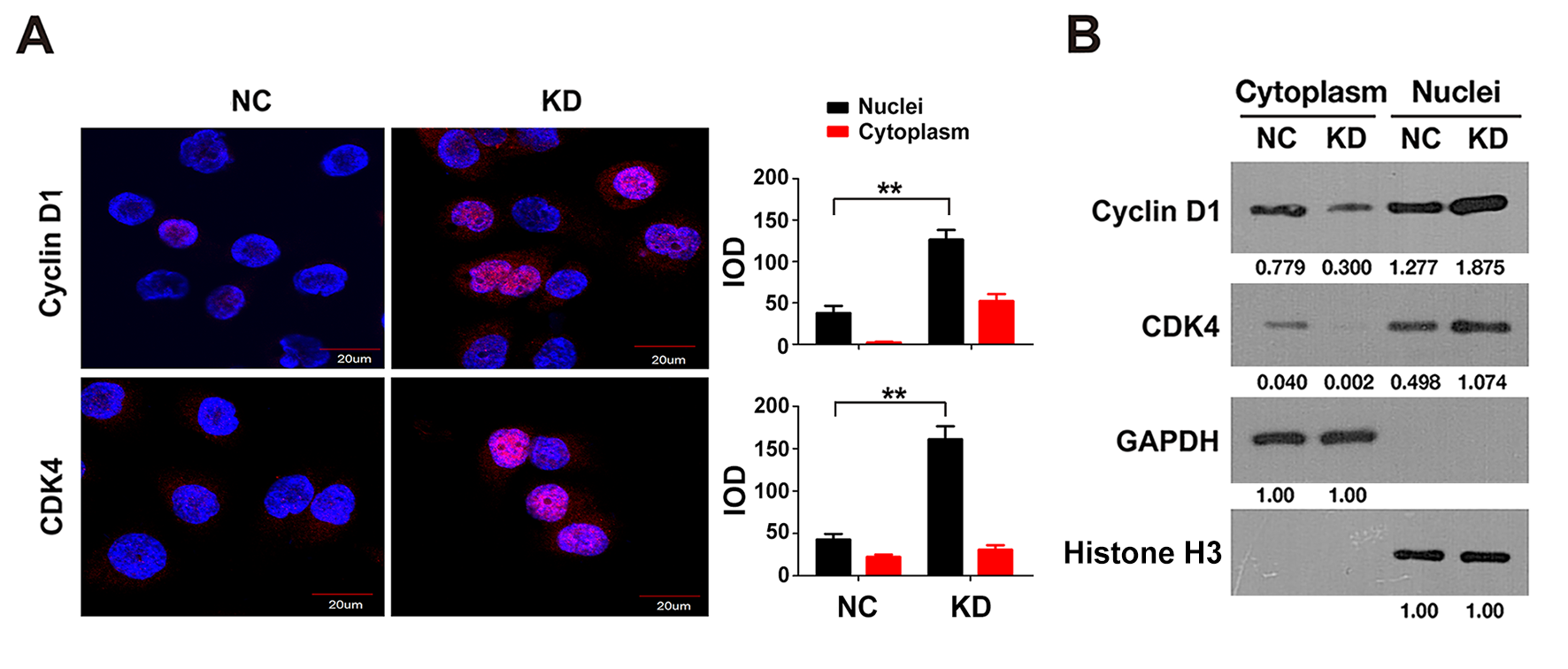

Supplement: Supplementary file 9 — Figure S6. RN181 knockdown increases translocation of cyclin D1 and CDK4 from the cytoplasm to the nuclei of AGS cells. [file PATH-248-204-s008.tif]

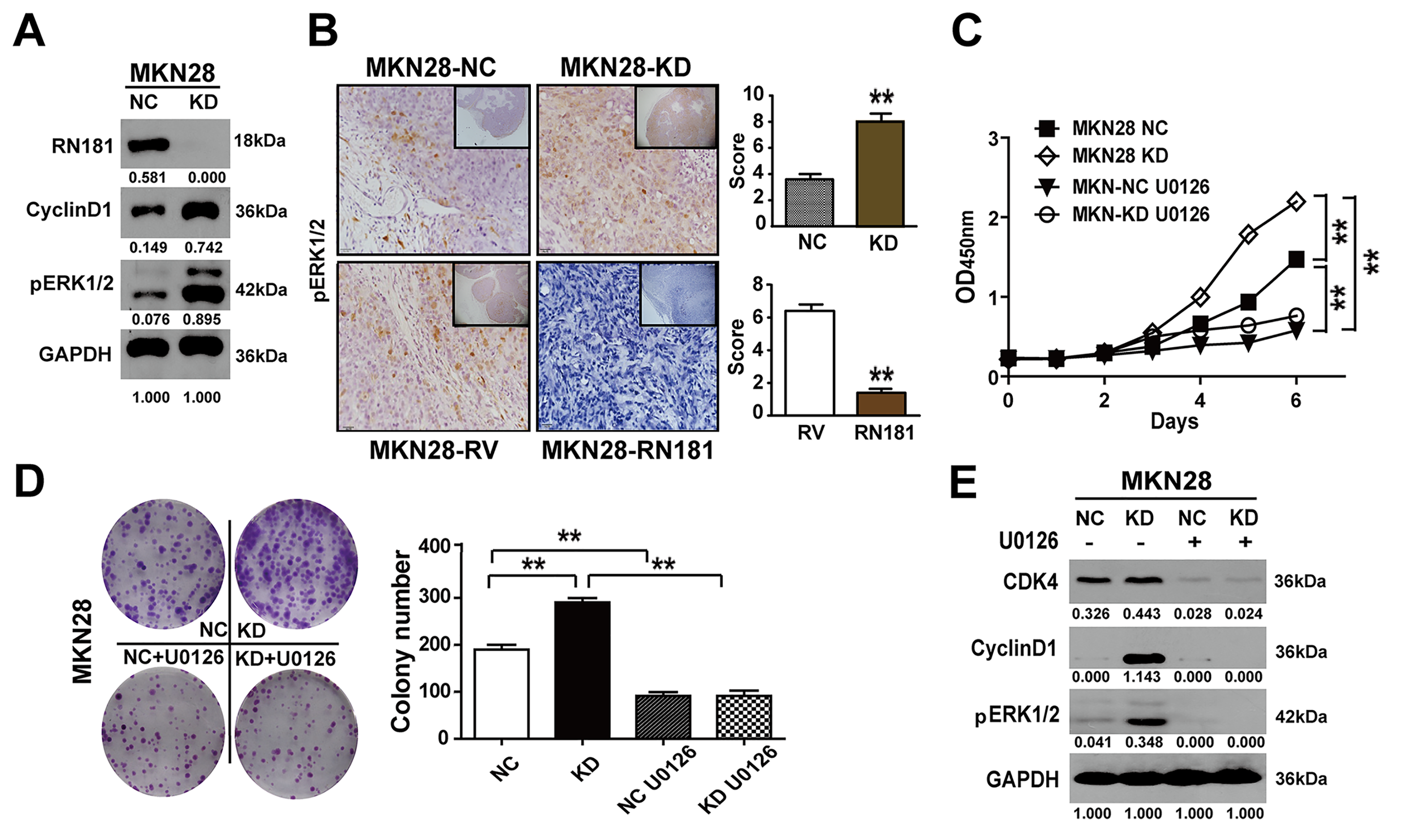

Supplement: Supplementary file 10 — Figure S7. RN181 suppresses tumour growth by inhibition of ERK/MAPK signalling in MKN28 cells. [file PATH-248-204-s009.tif]
